# Stabilization of Cardiogenic Shock for Critical Care Transport, a Simulation

**DOI:** 10.21980/J82354

**Published:** 2025-04-30

**Authors:** Matthew Heffernan, Jennifer Quinn, Craig Tschautscher, Ryan Newberry, Andrew Cathers, Brittney Bernardoni

**Affiliations:** * University of Wisconsin-Madison School of Medicine and Public Health, Department of Emergency Medicine, Madison, WI; ^ UW Med Flight, Madison, WI

## Abstract

**Audience:**

This simulation is designed for critical care transport providers but can be easily adapted for the inpatient setting. It is applicable to an interdisciplinary team including nurses, respiratory therapists, medical students, emergency medicine residents, and emergency medicine attendings.

**Introduction:**

Cardiogenic shock carries an incredibly high burden of morbidity and mortality. Acute myocardial infarction accounts for 81% of cardiogenic shock patients and is a common indication for transfer to a tertiary care facility.^1^ Hypotension due to cardiogenic shock is often refractory to volume resuscitation and often requires pharmacologic intervention. Additionally, the resultant end organ dysfunction frequently requires advanced ventilatory support.^1^–^6^ This simulation aims to educate critical care transport providers on the best practices for management of the cardiogenic shock patients requiring resuscitation and intubation prior to transport.

**Educational Objectives:**

By the end of this simulation session, learners will be able to: 1) recognize the need for intubation in an unstable patient in cardiogenic shock who requires transport, 2) appropriately titrate bi-Level non-invasive ventilatory support (BiPAP) to optimize oxygenation and ventilation in preparation for intubation, 3) choose appropriate vasoactive medications to support the hemodynamics of a patient in cardiogenic shock, 4) perform rapid sequence intubation using appropriate induction and paralytic agents and dosing for a patient in cardiogenic shock, 5) choose appropriate initial lung-protective ventilator settings, and 6) implement an adequate analgesia and sedation plan for transport of an intubated patient in cardiogenic shock.

**Educational Methods:**

This session was conducted using high-fidelity simulation, allowing learners to manage a patient in cardiogenic shock and respiratory distress requiring intubation. Each session was followed by a debriefing and discussion.

**Research Methods:**

Qualitative feedback provided by participants during the discussion session was utilized to adjust the simulation between each session. In addition, participants were surveyed using a five-point Likert scale (strongly disagree to strongly agree) on if the simulation met their professional and educational needs, its efficacy and appropriateness for Level, and whether it would change future practice.

**Results:**

A total of 36 learners, including 20 physicians and 16 nurses, participated in the simulation over a total of nine sessions. Twenty out of the thirty-six participants completed the survey (both RNs and MDs) and 100% responded “strongly agree” to all four prompts (top response out of a five Likert scale). Feedback provided by participants was used after each session to adjust the simulation. Changes implemented included the addition of a nurse confederate, greater emphasis on management and titration of non-invasive ventilation for optimal preoxygenation, and initiation of post intubation sedation and analgesia.

**Discussion:**

Cardiogenic shock is a common cause of mortality, often requires transport, and is particularly challenging to manage. This simulation was overall effective at educating learners on the resuscitation of cardiogenic shock, including appropriate use of vasopressors and ventilatory support.

**Topics:**

Cardiogenic shock, hypoxic respiratory failure, vasopressor management, airway management, intubation, non-invasive positive pressure ventilation management, ventilatory management, emergency medicine, critical care transport medicine.

## USER GUIDE

**Table:** 

List of Resources:	
Abstract	31
User Guide	33
Instructor Materials	36
Operator Materials	45
Debriefing and Evaluation Pearls	50
Simulation Assessment	53

**Learner Audience:**

Medical Students, Interns, Junior EM Residents, Senior EM Residents, Critical Care Transport Physicians, Critical Care Transport Nurses, Critical Care Transport Respiratory Therapists

**Time Required for Implementation:**

**Instructor Preparation:** 15–30 minutes

**Time for case:** 10 minutes

**Time for debriefing:** 10–15 minutes

**Recommended Number of Learners per Instructor:**

Two to three learners per case; a critical care transport physician, a critical care transport nurse, and a sending hospital bedside nurse confederate. Additional team members, including respiratory therapists, nurses, resident physicians, and/or medical students, can also be included and/or substituted.

**Topics:**

Cardiogenic shock, hypoxic respiratory failure, vasopressor management, airway management, intubation, non-invasive positive pressure ventilation management, ventilatory management, emergency medicine, critical care transport medicine.

**Objectives:**

By the end of this session, the learner should be able to:

1. Recognize the need for intubation in an unstable patient in cardiogenic shock who requires transport.
2. Appropriately titrate bi-Level non-invasive ventilatory support (BiPAP) to optimize oxygenation and ventilation in preparation for intubation.
3. Choose appropriate vasoactive medications to support the hemodynamics of a patient in cardiogenic shock.
4. Perform rapid sequence intubation using appropriate induction and paralytic agents and dosing for a patient in cardiogenic shock.
5. Choose appropriate initial lung-protective ventilator settings.
6. Implement an adequate analgesia and sedation plan for transport of an intubated patient in cardiogenic shock.

### Linked objectives and methods

This simulation requires a multidisciplinary team of learners to implement core resuscitative techniques to stabilize airway, breathing, and circulation, in the context of a physiologically challenging patient. The learners will use physical exam findings, objective data, and clinical acumen to identify the need for intubation (objective 1). The learners will then recognize the necessity for optimization of oxygenation and hemodynamics prior to intubation. This will require learners to utilize multidisciplinary skills to plan and implement the appropriate adjustments to BiLevel Positive Airway Pressure (BiPAP) settings (objective 2) and vasoactive medications (objective 3) in accordance with the patient’s clinical condition. Once appropriately resuscitated, the learners will then perform rapid sequence intubation, using modified dosing of induction and paralytic agents to avoid hypotension (objective 4). After intubation, learners will work as a multidisciplinary team to initiate appropriate ventilator settings (objective 5) and sedation and analgesia (objective 6) to help facilitate successful transport of the patient.

### Recommended pre-reading for instructor

The instructors for this course should familiarize themselves with the pathophysiology and guidelines for treatment of cardiogenic shock. A few recommended resources to review this topic include the 2019 review article on cardiogenic shock from the *Journal of the American Heart Association*^1^ and the 2024 review article on cardiogenic shock from the *Annals of Intensive Care*.^2^ Instructors should also be familiar with up-to-date guidelines on emergency airway management and resuscitation, with particular emphasis on the hemodynamically compromised airway. This topic is covered in depth in the 2015 *Critical Care Horizons* article, “Airway Management of the Critically Ill Patient: Modifications of Traditional Rapid Sequence Induction and Intubation,”^6^ and in *The Walls Manual of Emergency Airway Management.*^10^,

### Results and tips for successful implementation

This simulation was conducted as part of the University of Wisconsin Med Flight Simulation Education Series between 2021 and 2023. It was presented to several cohorts of critical care transport providers as part of a bi-monthly series of simulation days designed to hone teamwork, technical skills, and best practices. A total of 36 learners, including 20 physicians and 16 nurses, participated in the simulation over a total of nine sessions. The first eight sessions were conducted in 2021 over two days, two weeks apart and included groups of two physicians and two nurses. The last session was conducted in 2023 with four physician fellows, participating as part of their fellowship orientation curriculum, and four nurse educators.

After each implementation, learners participated in a debriefing session during which feedback on simulation content was collected. In addition, participants were surveyed using a five-point Likert scale (strongly disagree to strongly agree) on the following prompts:

- This simulation met my professional and educational needs
- The manner in which this material was presented was effective
- This simulation was appropriate for my professional licensure Level
- This simulation will change my future practice

Feedback was overall positive. Twenty out of the thirty-six participants completed the survey (both RNs and MDs), and 100% responded “strongly agree” to all four prompts (top response out of a 5 Likert scale). Critical feedback was utilized to adjust the simulation, which evolved over the nine sessions, eventually reaching its current form after implementation of feedback from the final session in 2023. Specific modifications added as a result of feedback include; the addition of nurse confederate prompts to help facilitate progression through the case, modification of vital signs including greater fluctuations in blood pressure and hypoxia to trigger learner responses, addition of BiPAP to the initial presentation, assessment of percent leak/BiPAP adequacy, and initiation of post intubation sedation.

## Supplementary Information



## Figures and Tables

**Table:** 

#1	Chest radiograph
#2	Electrocardiogram
#3	Point of care lab values

**Table:** 

**Vitals:**	Heart Rate (HR) 117	Blood Pressure (BP) 92/50
	Respiratory Rate (RR) 18	Temperature (T) 37.2°C
	Oxygen Saturation (O_2_Sat) 88%	
	Weight: 120kg	Height: 5’7” (ideal body weight ~75kg)

**Figure:**
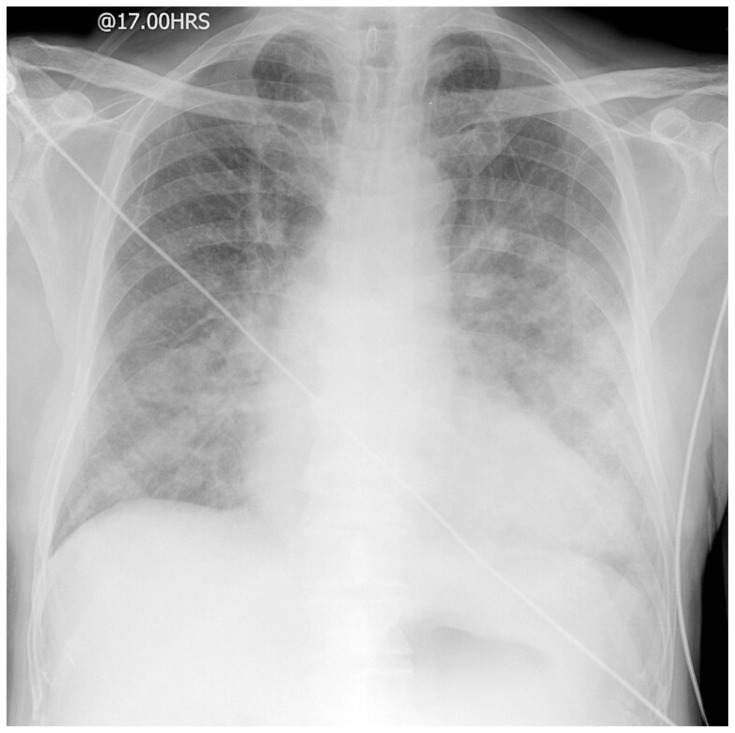


**Figure:**
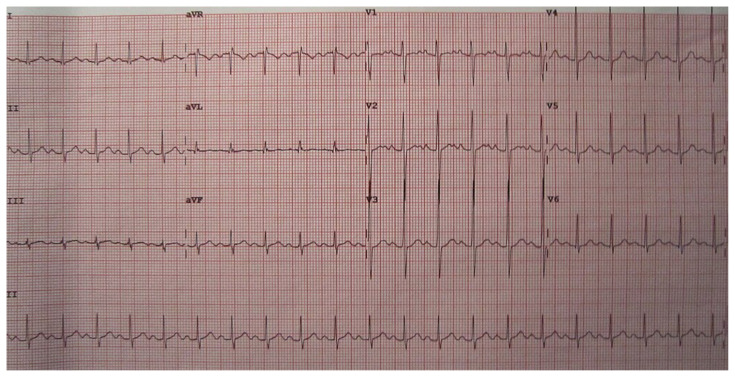


**Table:** 

*Point of care lab values:*	
pH	7.30
pCO2	37 mmHg
pO2	75 mmHg
Total CO2	17 mEq/L
Base excess	−5.2 mEq/L
O2 saturation	90%
Sodium	135 mEq/L
Potassium	4.7 mEq/L
Ionized Calcium	3.0 mg/dL (low)
Chloride	98 mEq/L
Bicarbonate	18 mEq/L
Anion Gap	19 mEq/L
Hemoglobin	9.4 g/dL
Glucose	112 mg/dL
Lactate	5 mmol/L
Creatinine	2.2 mg/dL

**Table:** SIMULATION EVENTS TABLE:

Minute (State)	Participant Action/ Trigger	Patient Status (Simulator Response) & Operator Prompts	Monitor Display (Vital Signs)
Stage 1 (Baseline)	**Learner objectives and interventions:** Place patient on monitor Obtain history and physical exam Assess BiPAP adequacy: ○ Identify hypoxia ○ Assess % leak on BiPAP mask ○ Assess tidal volume and delta pressure. Recognize need for intubation prior to transport Start resuscitation in preparation for intubation ○ Improve seal on BiPAP mask ○ Increase PEEP ○ Start vasopressor (norepinephrine preferred) ○ Consider inotropic agent (epinephrine or dobutamine) ○ Consider stopping dopamine ○ Push dose epinephrine/Ca available **Triggers:** - If learner starts to discuss/prepare to transport patient prior to intubation, give prompt from nurse. - If asks for point of care labs, give point of care labs stimulus. - If asks for chest x-ray, give chest x-ray stimulus. - If starts to discuss intubation prior to addressing oxygenation or hemodynamics, move to 2nd stage. - If starts norepinephrine or epinephrine and addresses BiPAP by improving mask seal and/or increasing PEEP, move to 3rd stage.	**Sounds/auscultations:** Diffuse rales bilaterally. Normal heart and abdominal sounds. **Patient responses:** Confused, one- word answers. **Appearance/PE:** Sitting upright in bed. Eyes open. Weak peripheral pulses. Loose BiPAP mask in place. **Current BiPAP settings:** IPAP: 20 EPAP: 10 FiO2: 100% TV: 320ml % leak: 45% **Prompt:** Bedside nurse: “This patient looks like he’s really struggling on BiPAP. Do you still think he’ll be safe to transport?”	T 37.2° C HR 117 BP 92/50 RR 18 O2 88%
Stage 2	**Learner objectives and interventions:** Identify the need for treating hypotension prior to intubation. Utilize the correct vasopressor for cardiogenic shock (norepinephrine as first line, epinephrine acceptable alternative). Identify need for optimizing BiPAP settings prior to intubation. ○ Improve seal on BiPAP mask ○ Increase PEEP **Triggers:** - If gives norepinephrine or epinephrine, move to 3rd stage. If norepinephrine or epinephrine is not given, vital signs do not improve.	**Appearance/PE:** Patient has had a slight drop in mean arterial pressure (MAP), now <65 **Prompt:** Bedside nurse: “The patient’s BP looks a little soft. Do we want to treat that?”	T 37° C HR 123 BP 88/50 RR 22 O2 86%
Stage 3	**Learner objectives and interventions:** Set-up for intubation ○ Discuss intubation strategy and plan (eg: standard geometry video laryngoscopy + bougie). Choose an appropriate induction agent (etomidate 0.1-0.2 mg/kg TBW vs ketamine 1-1.5 mg/kg IBW) and paralytic (rocuronium 1.5-2 mg IBW or succinylcholine 1.5-2 mg TBW. Perform successful RSI. **Triggers:** - If push dose norepinephrine or epinephrine is given with induction/intubation, move to 5th stage. - If just gives induction agent without norepinephrine or epinephrine, move to the 4th stage. - If no intubation is performed, prompt from the bedside nurse.	**Sounds/auscultation:** Diffuse rales bilaterally. Tachycardic. **Patient responses:** Confused, one- word answers. **Appearance/PE:** Eyes open. Improved peripheral pulses. **Prompt:** Bedside nurse: “Are you all ready to go with intubation?”	T 37° C HR 119 BP 95/54 RR 20 O2 90%
Stage 4	**Learner objectives and interventions:** Recognize the need for vasoactive medications to treat hypotension following induction. Choose appropriate vasopressors given the patient’s cardiogenic shock physiology (norepinephrine or epinephrine). **Trigger:** - If gives push dose norepinephrine/epinephrine or increases vasopressor drip, move to stage 5. - If just gives fluid, blood pressure does not change. - If no additional vasopressor is given, vital signs do not improve.	**Sounds/auscultation:** Diffuse rales bilaterally. Tachycardic. **Appearance/PE:** Intubated and sedated. Weak peripheral pulses.	T 37° C HR 120 BP 82/46 RR 20 O2 92%
Stage 5	**Learner objectives and interventions:** Place patient on lung-protective transport ventilator settings. ○ Tidal volume (TV) (6-8cc/kg), ○ PEEP ≥ 10 mmHg ○ Wean FiO2 for SpO2 > 88% ■ Lowest FiO2 to achieve this goal should be 50% if PEEP ≥ 10 mmHg ○ Assess plateau pressure ■ Set plateau pressure (PPlat) at 14 mmHg above PEEP if appropriate TV is chosen. Start post-intubation sedation and analgesia. **Trigger:** - If no sedation is started, give a prompt from the bedside nurse.	**Sounds/auscultation:** Diffuse rales bilaterally. Tachycardic. **Appearance/PE:** Intubated and sedated. Improved peripheral pulses. **Prompt:** Bedside nurse: “Do you want to start any sedation now?”	T 37° C HR 112 BP 97/56 RR 20 O2 92%
(Case Completion)	**Learner objectives and interventions:** Transduce arterial line. Transfer IV infusions to transport pumps. **Trigger:** - If start to transfer IV infusions to transport pumps, end case.		T 37° C HR 112 BP 97/56 RR 20 O2 92%

**Table:** 

**Critical Actions:** Identify the need for intubation. Address the patient’s hemodynamic instability prior to intubation with appropriate vasopressor support. Optimize patient oxygenation and ventilation prior to intubation by assessing adequacy of non-invasive ventilatory support settings, including mask seal, and escalating settings with increased PEEP (10+). Utilize appropriate induction agents for cardiogenic shock. This includes etomidate 0.1–0.2 mg/kg of TBW or ketamine 1–1.5 mg/kg of IBW. Use an appropriately dosed paralytic agent for cardiogenic shock. This includes rocuronium 1.5–2 mg/kg of IBW or succinylcholine 1.5–2 mg/kg of TBW. Choose lung-protective ventilator settings (tidal volume 6–8 cc/kg IBW, plateau pressure <30 mmHg, FiO2 less than or equal to 60%) Initiate post intubation sedation and analgesia.	0:00

**Table:** 

	Milestone	Did not achieve level 1	Level 1	Level 2	Level 3
1	**Emergency Stabilization (PC1)**	□ Did not achieve Level 1	□ Recognizes abnormal vital signs	□ Recognizes an unstable patient, requiring intervention Performs primary assessment Discerns data to formulate a diagnostic impression/plan	□ Manages and prioritizes critical actions in a critically ill patient Reassesses after implementing a stabilizing intervention
2	**Performance of focused history and physical (PC2)**	□ Did not achieve Level 1	□ Performs a reliable, comprehensive history and physical exam	□ Performs and communicates a focused history and physical exam based on chief complaint and urgent issues	□ Prioritizes essential components of history and physical exam given dynamic circumstances
3	**Diagnostic studies (PC3)**	□ Did not achieve Level 1	□ Determines the necessity of diagnostic studies	□ Orders appropriate diagnostic studies. Performs appropriate bedside diagnostic studies/procedures	□ Prioritizes essential testing Interprets results of diagnostic studies Reviews risks, benefits, contraindications, and alternatives to a diagnostic study or procedure
4	**Diagnosis (PC4)**	□ Did not achieve Level 1	□ Considers a list of potential diagnoses	□ Considers an appropriate list of potential diagnosis May or may not make correct diagnosis	□ Makes the appropriate diagnosis Considers other potential diagnoses, avoiding premature closure
5	**Pharmacotherapy (PC5)**	□ Did not achieve Level 1	□ Asks patient for drug allergies	□ Selects an medication for therapeutic intervention, consider potential adverse effects	□ Selects the most appropriate medication and understands mechanism of action, effect, and potential side effects Considers and recognizes drug-drug interactions
6	**Observation and reassessment (PC6)**	□ Did not achieve Level 1	□ Reevaluates patient at least one time during case	□ Reevaluates patient after most therapeutic interventions	□ Consistently evaluates the effectiveness of therapies at appropriate intervals
7	**Disposition (PC7)**	□ Did not achieve Level 1	□ Appropriately selects whether to admit or discharge the patient	□ Appropriately selects whether to admit or discharge Involves the expertise of some of the appropriate specialists	□ Educates the patient appropriately about their disposition Assigns patient to an appropriate level of care (ICU/Tele/Floor) Involves expertise of all appropriate specialists
9	**General Approach to Procedures (PC9)**	□ Did not achieve Level 1	□ Identifies pertinent anatomy and physiology for a procedure Uses appropriate Universal Precautions	□ Obtains informed consent Knows indications, contraindications, anatomic landmarks, equipment, anesthetic and procedural technique, and potential complications for common ED procedures	□ Determines a back-up strategy if initial attempts are unsuccessful Correctly interprets results of diagnostic procedure
20	**Professional Values (PROF1)**	□ Did not achieve Level 1	□ Demonstrates caring, honest behavior	□ Exhibits compassion, respect, sensitivity and responsiveness	□ Develops alternative care plans when patients’ personal beliefs and decisions preclude standard care
22	**Patient centered communication (ICS1)**	□ Did not achieve level 1	□ Establishes rapport and demonstrates empathy to patient (and family) Listens effectively	□ Elicits patient’s reason for seeking health care	□ Manages patient expectations in a manner that minimizes potential for stress, conflict, and misunderstanding. Effectively communicates with vulnerable populations, (at risk patients and families)
23	**Team management (ICS2)**	□ Did not achieve level 1	□ Recognizes other members of the patient care team during case (nurse, techs)	□ Communicates pertinent information to other healthcare colleagues	□ Communicates a clear, succinct, and appropriate handoff with specialists and other colleagues Communicates effectively with ancillary staff

## INSTRUCTOR MATERIALS

**Case Title:** Critical Care Transport Cardiogenic Shock Intubation Simulation

**Case Description & Diagnosis (short synopsis):** This scenario presents a 70-year-old gentleman suffering from a non-ST elevation myocardial infarction (NSTEMI) with resultant acute left heart failure exacerbation that requires transport to a tertiary care center. The learner arrives to find the patient in cardiogenic shock and hypoxemic respiratory failure. They will need to utilize appropriate resuscitative measures to stabilize him for transport. Key learning points include the appropriate use of vasopressors and assessment/titration of non-invasive positive pressure ventilation, which will be necessary to perform a successful rapid sequence intubation, following which appropriate lung protective ventilator settings and post intubation sedation must be initiated.

**Equipment or Props Needed:**

- High-fidelity patient simulation model
- Personal protective equipment
- Patient monitor with end-tidal CO2 and arterial pressure monitoring capabilities
- Pulse oximeter
- Blood pressure cuff
- Peripheral IV catheters and lines x2
- Arterial line set-up
- Central line set-up
- IV pole
- IV pump
- End-tidal CO2 monitoring equipment
- Medications:
  ○ vasopressors: epinephrine, norepinephrine, dopamine, dobutamine, vasopressin, phenylephrine induction meds: etomidate, ketamine
- paralytic meds: rocuronium, succinylcholine
- sedative meds: propofol, ketamine
- analgesic med: fentanyl, hydromorphone
- Airway Supplies:
  ○ nasal cannula, non-rebreather face mask; bag-valve mask; PEEP valve, laryngeal mask airway
  ○ (LMA), suction catheter and wall or portable suction, endotracheal tubes, direct laryngoscope blades, stylet, oxygen supply
- BiPAP mask and non-invasive ventilation set-up.
- Mechanical transport ventilator with tubing
- Transport IV pumps
- Stethoscope

**Actors needed:**

- Assistant to play the sending hospital bedside nurse

**Stimulus Inventory:**

**Background and brief information:** Patient presents to a small community emergency department (ED) today with complaints of chest pain and shortness of breath. He was admitted to the intensive care unit after his ED workup revealed a NSTEMI. Over the past few hours, he has developed hypotension in addition to hypoxia and pulmonary edema requiring escalation to BiPAP ventilation and initiation of a dopamine infusion. A formal echocardiogram today revealed a newly reduced ejection fraction of 20% and mild right ventricular dysfunction.

**Initial presentation:** Patient is seated upright with a BiPAP mask loosely fitted on his face, confused, tachypneic with increased work of breathing, tachycardic, cool extremities and weak peripheral pulses.

**How the scene unfolds:** The case begins with an ill-appearing patient who has increased work of breathing on BiPAP and borderline hypotension on a continuous dopamine infusion. The transport team will need to work through an assessment of the patient’s BiPAP adequacy, identifying the need for intubation prior to transport. They will then need to perform resuscitation, addressing the patient’s hypoxia and hemodynamic instability prior to rapid sequence intubation. This will require the learners to utilize appropriate vasoactive medications, optimize the patient’s non-invasive ventilation settings, and choose appropriate induction and paralytic agents and dosing to avoid worsening hemodynamic instability with intubation. Following intubation, they must choose appropriate post intubation sedation and analgesia.

**Critical actions:**

1. Identify the need for intubation.
2. Address the patient’s hemodynamic instability prior to intubation with appropriate vasopressor support.
3. Optimize patient oxygenation and ventilation prior to intubation by assessing adequacy of non-invasive ventilatory support settings, including mask seal, and escalating settings with increased positive end expiratory pressure (PEEP; minimum of 10 mmHg).
4. Utilize appropriate induction agents for cardiogenic shock. This includes etomidate 0.1–0.2 mg/kg of total body weight (TBW) or ketamine 1–1.5 mg/kg of ideal body weight (IBW).
5. Use an appropriately dosed paralytic agent for cardiogenic shock. This includes rocuronium 1.5–2 mg/kg of IBW or succinylcholine 1.5–2 mg/kg of TBW.
6. Choose lung protective ventilator settings (tidal volume 6–8 cc/kg IBW, plateau pressure <30 mmHg, FiO2 less than or equal to 60%).
7. Initiate post intubation sedation and analgesia.

**Case Title:** Critical Care Transport Cardiogenic Shock Intubation Simulation

**Chief Complaint:** 70-year-old male with a history significant for hypertension, hyperlipidemia, coronary artery disease requiring placement of three stents one year ago. He presented to the emergency department today with complaints of chest pain and shortness of breath. He was found to have an NSTEMI and was admitted to the intensive care unit. He had an echo earlier today that showed a newly reduced ejection fraction of 20% and preserved right ventricular function. He has become progressively more hypoxic and hypotensive, so he was placed on BiPAP and started on a dopamine infusion.

**Other information:**

Infusions: dopamine at 10mcg/kg/min

BiPAP settings: IPAP 20/EPAP10; FiO2 100%

- BiPAP mask should be loosely applied to create a high % leak
- Patient should be sitting in upright position in bed

Lines: Right radial arterial line; triple lumen catheter in right internal jugular vein; bilateral antecubital fossa peripheral intravenous lines

**General Appearance:** confused, anxious and ill-appearing

**Primary Survey:**

- **Airway:** Patent
- **Breathing:** Diffuse rales in all lung fields. Increased work of breathing
- **Circulation:** weak peripheral pulses, tachycardic, regular rhythm, cool extremities

**History:**

- **History of present illness:** 70-year-old male who presented to a rural ED 48 hours ago with left-sided chest pressure and shortness of breath while eating breakfast. His chest pressure was associated with left arm numbness, nausea, and diaphoresis. He waited six hours before presenting to the ED. His pain improved with nitroglycerine, and he was admitted to hospital after receiving 324 mg of aspirin, 600 mg of clopidogrel, and heparin infusion. Remainder of the history is limited by the patient’s altered mental status
- **Past medical history:** hypertension, hyperlipidemia, coronary artery disease with three prior stents placed one year ago, 30 pack year smoking history.
- **Past surgical history:** none
- **Patient’s medications:** continuous: dopamine at 10 mcg/kg/min, heparin at 10 units/kg/hr. Scheduled: aspirin 81 mg daily, clopidogrel 75 mg daily, atorvastatin 80 mg daily.
- **Allergies:** none
- **Social history:** none
- **Family history:** none

**Secondary Survey/Physical Examination:**

- **General appearance:** confused, anxious and ill-appearing
- **HEENT:**
  ○ **Head:** within normal limits
  ○ **Eyes:** within normal limits
  ○ **Ears:** within normal limits
  ○ **Nose:** within normal limits
  ○ **Throat:** within normal limits
- **Neck:** within normal limits
- **Heart:** tachycardic, audible S1/S2, no murmurs, rubs, clicks or gallops.
- **Lungs:** Diffuse rales in all lung fields. Increased work of breathing
- **Abdominal/GI:** within normal limits
- **Genitourinary:** within normal limits
- **Rectal:** within normal limits
- **Extremities:** cool extremities, weak peripheral pulses, dry, no peripheral edema.
- **Back:** within normal limits
- **Neuro:** Alert and oriented to self and location, confused, following commands in all four extremities.
- **Skin:** Cool extremities
- **Lymph:** within normal limits
- **Psych:** Anxious

*Chest radiograph*

Gaillard G, Jones J. AP portable CXR of a patient in acute pulmonary oedema in: In: Wikimedia Commons.

https://upload.wikimedia.org/wikipedia/commons/8/82/AP_portable_CXR_of_a_patient_in_acute_pulmonary_oedema.jpg. Published July 23, 2009. Accessed April 14, 2024. CC BY-SA 3.0.

*Electrocardiogram:*

Heilman J, Sinus tachycardia as seen on ECG in: Wikimedia commons.

https://commons.wikimedia.org/wiki/Category:ECG_of_sinus_tachycardia#/media/File:Sinustachy.JPG. Published June 15, 2012. Accessed March 11, 2025. CC BY-SA 3.0.

## OPERATOR MATERIALS

**Diagnosis:**

Cardiogenic shock and hypoxemic respiratory failure.

**Disposition:**

Once intubated and stabilized, continue with transport to the tertiary care center.

## DEBRIEFING AND EVALUATION PEARLS

### Stabilization of Cardiogenic Shock for Critical Care Transport

**Pearls:**

1. **Hemodynamics should be optimized with use of vasopressors in the hypotensive cardiogenic shock patient.**
  - Norepinephrine or epinephrine are the preferred agents for treatment of hypotension in cardiogenic shock given their selective alpha and beta agonism, with a preference for norepinephrine.^1^–^2^ These agents should be utilized to maintain a mean arterial pressure >65 mmHg.^1^ This is of particular importance in patients who require intubation, given the negative hemodynamic effects associated with use of induction agents and positive pressure mechanical ventilation.^6^,^12^ Fluid administration may be ineffective and potentially harmful for patients in cardiogenic shock given frequent pre-existing venous congestion and potential for third spacing of fluid into the lungs and interstitial space leading to worsening hypoxia. As a result, recommendations regarding its use are varied. One approach offered by the American Heart Administration includes considering a small fluid challenge of 250–500 mL if hypovolemia is suspected, with a careful assessment of response.^1^–^4^
2. **Careful selection of induction and paralytic agents and adjustments in dosing, with preference for low dose sedative and higher doses of paralytic agents, can be considered in cardiogenic shock patients requiring rapid sequence intubation (RSI).**
  - The cardiogenic shock state poses a particular challenge with RSI given the threat of hemodynamic collapse. Sedative agents may induce vasodilation and blunting of the sympathomimetic drive. This can lead to decreased diastolic blood pressure and thus coronary perfusion pressure, thereby worsening/inducing cardiac ischemia and decreasing cardiac output further.^3^,^6^ As a result of this risk, careful selection and administration of sedative agents is imperative when performing RSI in the cardiogenic shock patient. While there is no concrete evidence to support one agent over another in cardiogenic shock, ketamine and etomidate are generally considered reasonable options due to their lesser effects on cardiac output and peripheral vascular resistance with low to standard dosing.^6^–^10^ Furthermore, some clinicians consider utilizing a lower dose of sedative in the shock state because this may further lower the risk of hemodynamic decompensation, and shocked patients may have preexisting decreased awareness due to decreased cerebral perfusion and metabolic derangements.^10^
  - Paralysis is utilized in RSI to maximize the likelihood of successful intubation and minimize apneic time. This is of particular importance in the cardiogenic shock patient whose tolerance of apnea may be negatively affected by their shock state.^6^ Additionally, given the low cardiac output state present in cardiogenic shock, a longer time may be needed for an intravenously administered paralytic to circulate through the vasculature, thus potentially increasing the time from paralytic administration to effect. As a result, some clinicians consider administering higher doses of paralytic for cardiogenic shock patients to achieve a more rapid onset of paralysis and thus decrease apneic time.^13^–^15^
  - In unstable patients who require intubation, including the cardiogenic shock patient, there are alternative techniques available for intubation without the use of sedative or paralytic agents. This includes the topicalized awake intubation, during which intubation is achieved using topicalized anesthetics while the patient maintains airway reflexes and spontaneous respiration. While this can be a highly effective method of intubation, evidence supporting its use in the emergency department, prehospital setting, and transport environment is very limited.^11^
3. **Bi-Level positive airway pressure (BiPAP) can be used to optimize preoxygenation and ventilation and requires an assessment of mask seal.**
  - BiPAP is a highly effective method of non-invasive ventilation that can improve both hypercarbia and hypoxia.^10^
  - Oxygenation is optimized by titrating expiratory positive airway pressure (EPAP) and fraction of inspired oxygen (FiO2). Increases in EPAP may lead to improved alveolar recruitment while increases in FiO2 increase the total amount of oxygen delivered with each breath, both resulting in more efficient oxygen delivery.^10^
  - Ventilation is optimized by titrating the difference between inspiratory positive airway pressure (IPAP) and EPAP (commonly referred to as the delta pressure) as well as respiratory rate. The delta pressure is one determinant of tidal volume (volume of air delivered with each breath). Higher delta pressure results in greater tidal volume, which in turn increases the amount of carbon dioxide exhaled with each breath. Higher respiratory rate increases the amount of volume exchanged over time, thereby further increasing carbon dioxide elimination.^10^,^12^
  - BiPAP is delivered through a mask which is inherently susceptible to leakage of air. The amount of leak is primarily dependent on the competence of the seal between the mask and the patient’s face. The percent leak (percentage of tidal volume escaping out of the system) can have profound effects on the amount of support provided by BiPAP, potentially decreasing the delivered EPAP, IPAP, FiO2 and tidal volume. Adequate mask seal therefore requires close assessment and attention in patients on BiPAP.^10^

**Other debriefing points:**

During debriefing, learners often reported feeling like they were missing something in regards to the patient’s hypoxia. Learners were expecting and looking for a higher patient oxygen saturation prior to intubation. While optimizing preoxygenation is clearly a focus of this sim, one of the key takeaways is that in real life you often can’t achieve an oxygen saturation of 100% due to underlying patient physiology. Inability to fully pre-oxygenate is a risk factor for hemodynamic decompensation and hypoxia during intubation attempts and should inform the peri-intubation resuscitation and stabilization plan.

## SIMULATION ASSESSMENT

*Stabilization of Cardiogenic Shock for Critical Care Transport*

Learner: _________________________________________

***Assessment Timeline***

This timeline is to help observers assess their learners. It allows observer to make notes on when learners performed various tasks, which can help guide debriefing discussion.

**Critical Actions:**

■ Identify the need for intubation.
■ Address the patient’s hemodynamic instability prior to intubation with appropriate vasopressor support.
■ Optimize patient oxygenation and ventilation prior to intubation by assessing adequacy of non-invasive ventilatory support settings, including mask seal, and escalating settings with increased PEEP (10+).
■ Utilize appropriate induction agents for cardiogenic shock. This includes etomidate 0.1–0.2 mg/kg of TBW or ketamine 1–1.5 mg/kg of IBW.
■ Use an appropriately dosed paralytic agent for cardiogenic shock. This includes rocuronium 1.5–2 mg/kg of IBW or succinylcholine 1.5–2 mg/kg of TBW.
■ Choose lung-protective ventilator settings (tidal volume 6–8 cc/kg IBW, plateau pressure <30 mmHg, FiO2 less than or equal to 60%)
■ Initiate post intubation sedation and analgesia.

**Summative and formative comments:**

**Milestones assessment:**
