# Supplementary material for: Stabilization of Cardiogenic Shock for Critical Care Transport, a Simulation
Source: J Educ Teach Emerg Med. 2025 Apr 30;10(2):S31–57. doi: 10.21980/J82354 (PMC12054092; doi:10.21980/J82354)
Supplement: Supplementary file 1 [file 10-2-S31-supp1.pptx]

## Slide 1
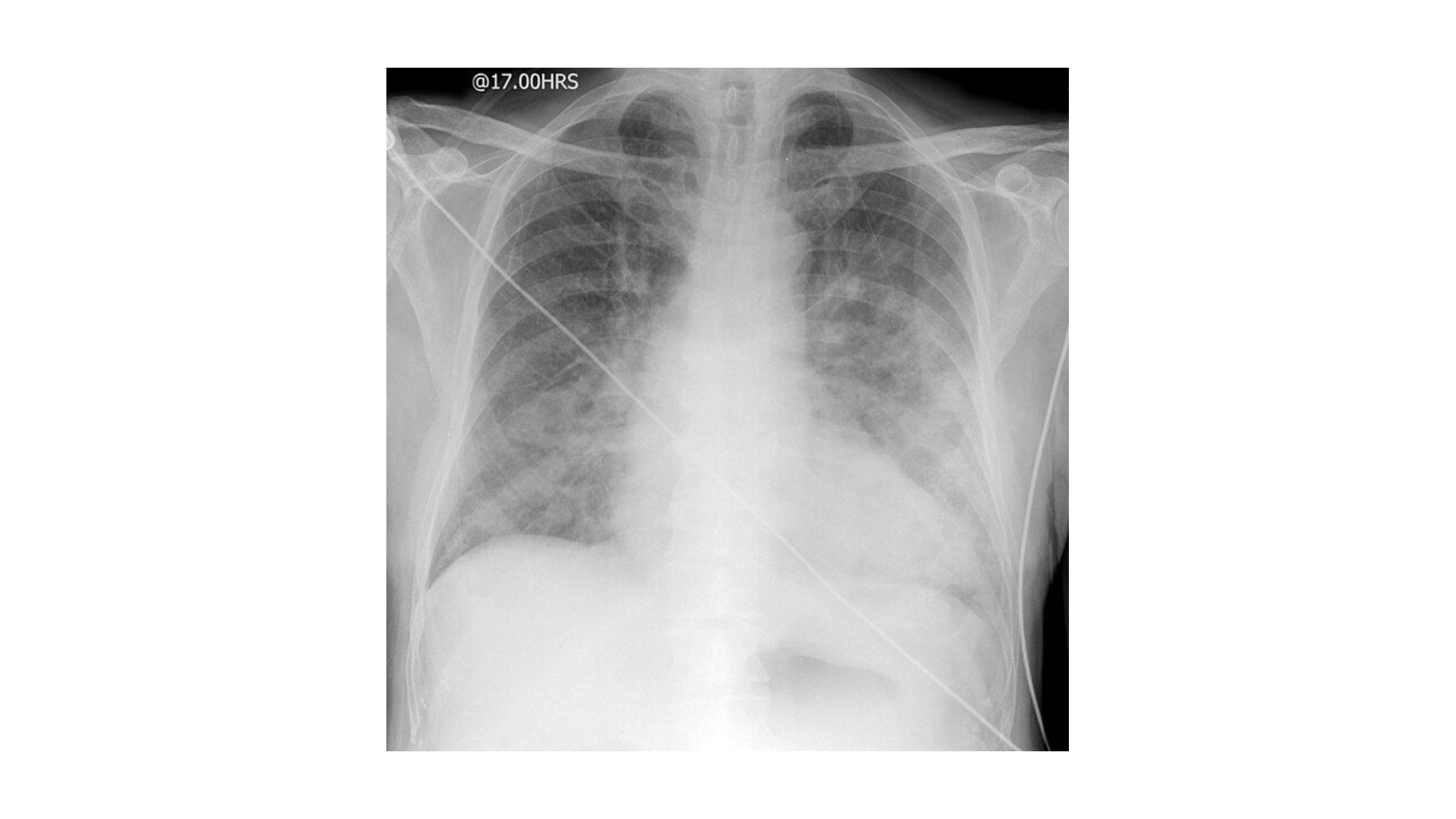

## Slide 2
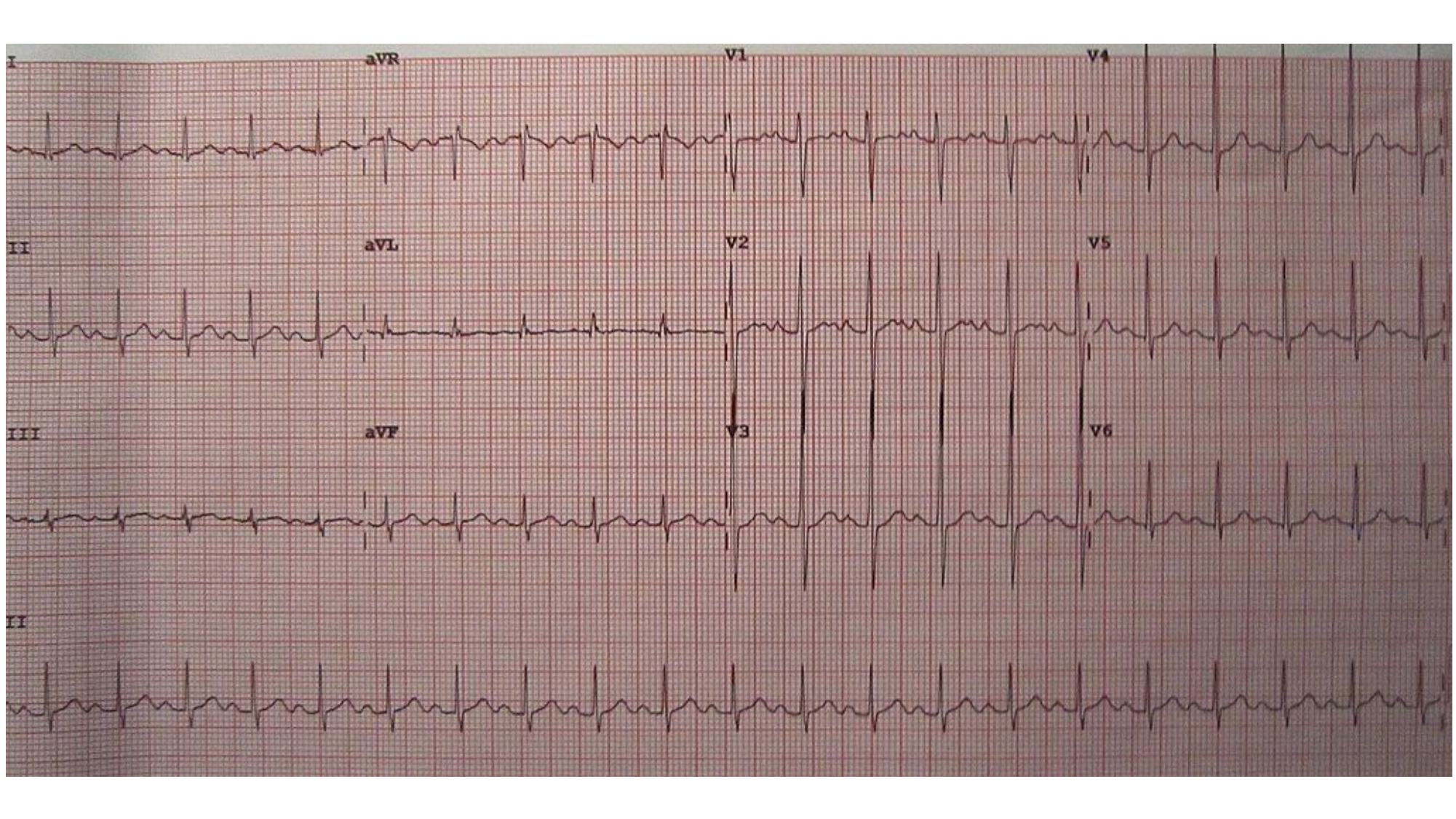

## Slide 3
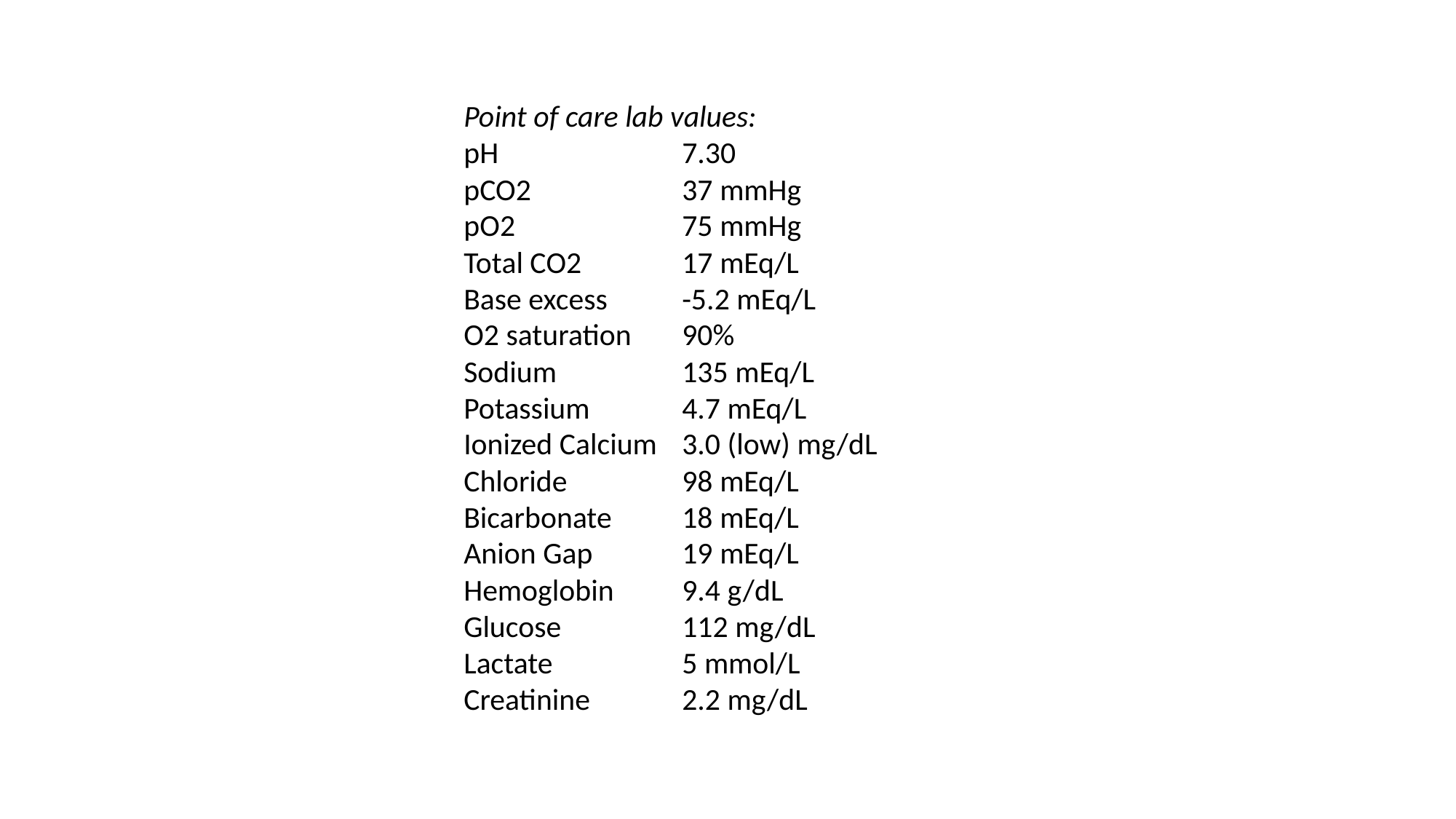

Point of care lab values:
pH 		7.30
pCO2		37 mmHg
pO2 		75 mmHg
Total CO2 	17 mEq/L
Base excess 	-5.2 mEq/L
O2 saturation	90%
Sodium		135 mEq/L
Potassium	4.7 mEq/L
Ionized Calcium	3.0 (low) mg/dL
Chloride		98 mEq/L
Bicarbonate	18 mEq/L
Anion Gap	19 mEq/L
Hemoglobin	9.4 g/dL
Glucose		112 mg/dL
Lactate 		5 mmol/L
Creatinine	2.2 mg/dL
